# Elements of Medical Chemistry

**Published:** 1866-11

**Authors:** 


For sale by W. B. Keen & Co., 148 Lake Street.

Elements of Medical Chemistry. By B. Howard Rand, M.D., Prof. of Chemistry in the Jefferson Medical College. Philadelphia: T. Edward Zell & Co., 17 & 19 South Sixth Street. 1867.

This is a neat little volume of 400 pages, being made up of a syllabus, or full notes, of the course of lectures given by the author in the Jefferson Medical College. The arrangement of the work is as follows:—An Introduction on the Properties and Qualities of Matter, and of Force. Part 1st. On Physics, including Gravitation, Light, Heat, and Electricity. Part 2d. On the Principles of Chemistry. Part 3d. On the Chemistry of the Elements. Part 4th. On Organic Chemistry. And an Appendix, on Weights and Measures; Incompatibility; Antidotes and Precautions in Medico-Legal Examinations; Precautions for Practice; List of Minerals; and Glossary. We think it will be found a most convenient little book of reference, both for students and practitioners.

